# Comparative effectiveness of acupoint stimulation for preventing postoperative nausea and vomiting after general anesthesia: a network meta-analysis of randomized trials

**DOI:** 10.1097/JS9.0000000000001976

**Published:** 2024-09-19

**Authors:** Ting Zhou, Huaijin Hou, Zhuoma Cairen, Yun Wang, Peng Wang, Long Ge, Macuo Wa, Ziqing Xu, Feng Tang, Caihong Wang, Rongxin Liu, Deyan Li, Jianjun Xue, Senbing Zhang

**Affiliations:** a Department of Anesthesiology, Jingmen People’s Hospital, Jingmen, Hubei; b Anesthesia and Pain Medical Center, Gansu Hospital of Traditional Chinese Medicine, Lanzhou, Gansu; c Department of Anesthesiology, Qinghai Provincial People's Hospital, Xining, Gansu; d Department of Information, Zhongxiang Hospital of Traditional Chinese Medicine, Jingmen, Hubei; e Evidence-based Social Science Research Center, School of Public Health, Lanzhou University, Lanzhou, Gansu; f The First Clinical Medical College, Gansu University of Chinese Medicine, Lanzhou, Gansu; g Department of Anesthesiology, Sir Run Run Shaw Hospital, School of Medicine, Zhejiang University, Hangzhou, Zhejiang, China

**Keywords:** acupoint, general anesthesia, network meta-analysis, postoperative nausea and vomiting

## Abstract

**Objective:**

The objective was to systematically evaluate the effectiveness of different acupoint stimulation techniques in preventing postoperative nausea and vomiting (PONV) after general anesthesia.

**Methods:**

The authors searched PubMed, Cochrane Library, Web of Science, and Embase for relevant papers, about the effect of acupoint stimulation for preventing PONV from their inception to 31 July 2023. Two reviewers performed study screening, data extraction, and risk of bias assessment. The authors focused on patient important outcomes, including the incidence of PONV, postoperative nausea (PON), or postoperative vomiting (POV), and the number of patients requiring antiemetic rescue. The authors conducted network meta-analyses to estimate the relative effectiveness between different acupoint stimulation using Stata 17.0 and Revman 5.3 software.

**Results:**

The authors included 50 randomized trials involving 7372 participants (median age: 43.5 years, female: 73.3%). The network meta-analysis revealed that compared with the control (sham acupoint stimulation or blank control), antiemetic alone could significantly reduce the incidence of POV (RR 0.49, 95% CI: 0.36–0.69), but could not significantly reduce the incidence of PONV and PON (RR 0.49, 95% CI: 0.36–0.69; RR 0.81, 95% CI: 0.59–1.10; respectively); both TEAS and electroacupuncture alone significantly reduced the incidence of PONV, PON, and POV, and combined with antiemetic was usually more effective than single acupoint stimulation.

**Conclusions:**

Both TEAS and electroacupuncture, with or without antiemetic, could significantly reduce the incidences of postoperative nausea and vomiting after general anesthesia.

## Introduction

Highlights

- We conducted a network meta-analysis to generate a clinically useful ranking of acupuncture stimulation (with or without antiemetic drugs) by comparing different acupuncture stimulation techniques.
- Both TEAS and electroacupuncture, with or without antiemetic, could significantly reduce the incidences of postoperative nausea and/or vomiting after general anesthesia.
- Acupuncture and acupressure are examples of physical stimulation, while TEAS and electroacupuncture are examples of electrical stimulation. Electrical stimulation was more effective than physical stimulation in preventing postoperative nausea and/or vomiting, and combination with antiemetics was the most effective strategy.

Postoperative nausea and vomiting (PONV) refers to the symptoms of postoperative nausea (PON) and postoperative vomiting (POV) occurring within 24–72 h after surgery, where PON includes stomach discomfort, upper abdominal discomfort, or the urge to vomit, and POV involves a strong gag reflex along with the expulsion of gastric contents through the mouth^1^. Postoperative nausea and/or vomiting is one of the most common complications after general anesthesia, volatile anesthetics, nitrous oxide, opioid analgesia, and the duration of anesthesia are anesthesia risk factors for PONV^1,2^, most commonly occurring on the first postoperative day^3^, which can lead to increased intra-abdominal pressure in patients, increasing the risk of aspiration, bleeding, and wound dehiscence^4,5,1,2^. The incidence of PONV after general anesthesia is 30%, and the incidence is as high as 60–80% in patients at high-risk for PONV^3^. Antiemetics are commonly used in the prevention and treatment of PONV, patients with high-risk PONV are advised to take a combination of at least three to four different antiemetics^1,6^. These medications can, however, have negative effects that limit their usage in some situations, including headaches, irritability, dry mouth, low blood pressure, and cardiovascular problems. These problems can be avoided using nonpharmacological therapy^7^. Therefore, nonpharmacological techniques for the prevention of PONV are gradually gaining attention.

Acupoint stimulation therapy is nonpharmacological therapy that includes transcutaneous electrical acupoint stimulation (TEAS), acupuncture, auricular acupuncture, acupressure, electroacupuncture^8–11^. According to traditional Chinese medicine, surgery disrupts the body’s state of equilibrium by disrupting the movement of qi (energy flow) and blood, causing stomach qi to rise, causing nausea, and vomiting^12^. The function of the stomach is regulated by PC6 acupoint stimulation to reduce the reflux of qi, which can prevent nausea and vomiting^12^. In addition to PC6, other effective acupoints for treating PONV include LI4, ST36, BL10-11, BL18-26, SP4, SP6, ST34, and ST44^8,13^. Acupressure and TEAS are noninvasive and convenient procedures^14,15^. In comparison with acupuncture, the advantage of electroacupuncture is that it retains the effect of traditional acupuncture treatment on the basis of the addition of acupuncture points that are generated by the combination of electrical and physical stimulation^16^. The advantage of TEAS is that it is not invasive and is more acceptable to patients, and the disadvantage is that only electrical stimulation can be applied without the physical stimulation of a needle piercing the skin^15^. A growing number of randomized controlled trials (RCTs) have shown that acupoint stimulation alone or in combination with antiemetic drugs can reduce the incidence of PONV and the number of patients requiring antiemetic rescue, with few adverse events^8,9^. Previous studies have been limited to direct comparisons of two interventions, making indirect comparisons impossible, but also making it difficult to compare and link three or more interventions^17^.

Network meta-analysis (NMA) can be used to estimate the relative effects of three or more interventions, resulting in more accurate estimates than a single direct or indirect estimate, and to rank and grade the interventions being compared^18^. Therefore, we conducted an NMA to generate a clinically useful ranking of acupuncture stimulation (with or without antiemetic drugs) by comparing different acupuncture stimulation techniques in both direct and indirect manners to provide patients and physicians with clear information to guide clinical decision making.

## Materials and methods

### Search strategy

The study had been registered in the International Prospective Register of Systematic Reviews (PROSPERO) (www.crd.york.ac.uk/PROSPERO), registration number. The work has been reported in line with Preferred Reporting Items for Systematic Reviews and Meta-Analyses (PRISMA) (Supplemental Digital Content 1, http://links.lww.com/JS9/D439, Supplemental Digital Content 2, http://links.lww.com/JS9/D440) and assessing the methodological quality of systematic reviews (AMSTAR) (Supplemental Digital Content 3, http://links.lww.com/JS9/D441) Guidelines^19,20^.

We searched PubMed, Cochrane Library, Web of Science, and Embase for relevant papers about the effect of acupoint stimulation for preventing PONV from 1 January 2000 to 31 July 2023. The search terms included terms related to acupoint stimulation (‘transcutaneous electrical acupoint stimulation’ OR ‘TEAS’ OR ‘electroacupuncture’ OR ‘acupuncture’ OR ‘acupressure’) and terms related to postoperative nausea and vomiting (‘postoperative nausea and vomiting’ OR ‘PONV’ OR ‘PON’ OR ‘POV’). There are no limitations on date, sex, or type of procedure. We retrieve the relevant literature by searching for these terms in the titles and abstracts.

### Study selection and eligibility criteria

Two independent reviewers screened the titles and abstracts of the articles. The full texts of eligible articles were then separately reviewed by the same two reviewers.

The inclusion criteria for the study were as follows: (1) adult patients who are under general anesthesia and have POV, PON, or POV after operation; (2) the acupoint stimulation techniques used in the study included transcutaneous electrical acupoint stimulation (TEAS), electroacupuncture, acupoint pressing, acupuncture, etc., with or without antiemetic drugs. The control group was blank control or sham acupoint stimulation; (3) RCTs; (4) studies reporting outcome data on the incidence of PONV, postoperative nausea (PON), postoperative vomiting (POV), and the number of patients requiring rescue antiemetic; (5) articles published in English.

The exclusion criteria for the study were as follows: (1) chemotherapy-induced nausea and vomiting; (2) studies combining two or more acupoint stimulation techniques; (3) studies without the description of the acupoint implemented; (4) descriptive studies; (5) literature on various experiences, case presentations, reviews, animal experiments, conference materials, etc.; (6) repeated published studies.

### Risk of bias assessment

Two trained researchers independently evaluated the risk of bias in the included literature according to the Cochrane Risk of Bias Assessment Tool recommended by the Cochrane Handbook for Systematic Reviews^21^, which contains the following criteria: (1) generation of random sequences; (2) allocation concealment; (3) blinding of participants and implementers; (4) blinding of outcome evaluations; (5) incomplete outcome data; (6) selective reporting; and (7) other biases. The result of each criterion was judged as ‘high-risk’, ‘unclear’, or ‘low risk’ by analyzing the included literature. Two researchers independently cross-checked the results, and if there was disagreement, a third researcher was asked to discuss the decision together.

### Data extraction

The data extraction process was performed independently by both authors. The basic information extraction form was developed, and the following information was extracted from each study: name of the first author, publication year, country, sample size, type of surgery, interventions, and outcomes. The data in the images was extracted by Engauge Digitizer software. We resolve differences through discussion or consultation with the third author.

### Statistical analysis

Statistical analysis was performed using Stata 17.0 and Revman 5.3 software for network meta-analysis. Dichotomous data were assessed using relative risks (RRs) and 95% CIs. We evaluated network heterogeneity for all treatment comparisons using the *I* ^2^ statistic and loop-specific heterogeneity using the τ^2^ statistic. We presented a network graph depicting direct comparisons between various interventions, with the size of the nodes indicating the sample size for each intervention and the thickness of the continuous line connecting the nodes denoting the number of studies directly comparing two interventions. When there was a closed loop, the consistency test was carried out by the node analysis method. If the difference was not statistically significant (*P*>0.05), it indicated that the consistency was good, and the consistency model was used for analysis, and the results were ranked; otherwise, the inconsistency model was used for analysis. We utilized a random effects model through the Stata software. The greater the area under the curve (surface under the cumulative ranking, SUCRA) by probability cumulative ranking, the greater the likelihood that an intervention was more effective. The risk of publication bias was assessed through the implementation of a correction comparison funnel. All tests were bilateral, and the test level was α=0.05.

## Results

### Literature search

**Figure 1:**
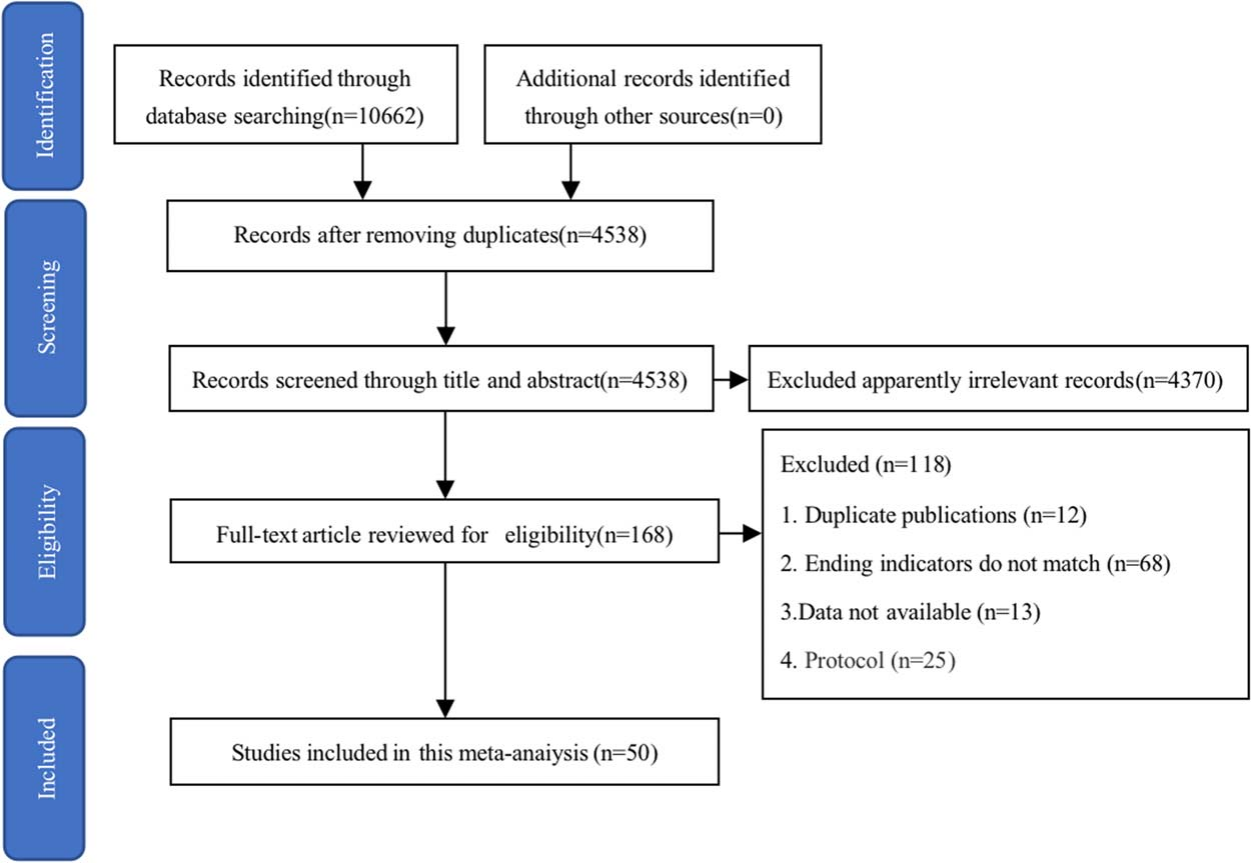
Flow diagram of study selection.

The flow chart of study selection is shown in Figure 1. Finally, 50 studies^22–53^ were included in this meta-analysis.

### Characteristics of included studies

A total of 7372 participants were included in the 50 trials, with 3748 being in the treatment group and 3624 being in the control group. About 50% of operations were laparoscopic and 72% were abdominal. The median age was 43.5 years [interquartile range (IQR): 30.5–56.5], and the median proportion of women was 73.3% (50–100%). Among the included randomized controlled trials, TEAS was the intervention in 15 studies, electroacupuncture in 9, acupressure in 18, and acupuncture in 8. The detailed characteristics of the included studies are shown in Table 1.

**Table 1:** Characteristics of the included trials.

					Intervention			
References	Country	Surgery	Age (T/C)	Number of participants (T/C)	T	C	Time point	Outcomes
Agarwal A^22^	India	Endoscopic surgery in Urology	n1:36.5±8.2 n2:34.2±6.1	100/100	Acupressure	Control	Before anesthesia begins	①
Zárate E^23^	NA	Laparoscopic cholecystectomy	n1:42± 16 n2:43±16	110/55	TEAS	Control	5–10 min before the end of the operation, wear for 9 h	②③④
Agarwal A^24^	India	laparoscopic cholecystectomy	n1:39.2±10.4 n2:41.4±12.3 n3:40.2± 9.9	50/50/50	Acupressure	Antiemetic; Control	30 min before anesthesia induction	②③④
Coloma M^25^	America	Laparoscopic surgery	n1:42±15 n2:42±16 n3:35±9	30/30/30	TEAS+Antiemetic	TEAS; Antiemetic	NA	②③
Ming JL^26^	China	Nasal Endoscopic Surgery	NA	100/50	Acupressure	Control	1 h before surgery and 10–24 h after surgery	②③
White PF^27^	America	Plastic Operation	n1:45 ±11 n2:43 ±13 n3:46 ± 11	40/40/40	Acupressure+Antiemetic	Acupressure; Antiemetic	Wristband worn for 72 h after admission to PACU	①②③④
Samad K^28^	Pakistan	laparoscopic cholecystectomy	n1:38.16±8.82 n2:40.60±11.11	25/25	Acupressure	Control	30 min before induction of anesthesia to 6 h postoperatively	①
Schultz AA^29^	America	Gynecological Surgery	n1:44.0±8.1 n2:47.7±12.3	24/25	Acupressure	Control	NA	②
Gan TJ^30^	America	Breast Surgery	n1:44±12 n2:47±10 n3:47±12	26/25/24	Electroacupuncture	Antiemetic; Control	At least 30 min before anesthesia until the end of surgery	③④
Klein AA^31^	America	Cardiac Surgery	n1:62±10 n2:63±8	75/77	Acupressure	Control	Worn before induction of anesthesia to 24 h after extubation	②④
Streitberger^32^	Germany	Gynecological Surgery	n1:44.3±13.47 n2:46.5±13.4	105/106	Acupuncture	Control	20 min before induction of anesthesia or after anesthesia for 20 min	②③
Amir SH^33^	India	Middle Ear Surgery	n1:17.95±8.25 n2:21.10±7.48	20/20	Electroacupuncture	Control	20 min before induction of anesthesia	①④
Sharma S^34^	India	Laparoscopic cholecystectomy	n1:47.34+6.9 n2:46.69±7.5 n3:45.41±6.4	20/20/20	Acupuncture+Antiemetic	Acupuncture; Antiemetic	5 min before anesthesia induction, lasting 30 min	②③④
liu YY^35^	China	Laparoscopic cholecystectomy	n1:42±18 n2:40±19	48/48	TEAS	Control	At least 30 min before anesthesia induction, no more than 60 min, until the end of the operation	②③④
Sadighha A^36^	Iran	Laparoscopic cholecystectomy	n1:44.4±6.6 n2:44.4±6.86 n3:45±7.32	53/51/52	Acupressure	Antiemetic; Control	NA	①②③
Korinenko Y^37^	America	Coronary artery bypass grafting and/or heart valve surgery	n1:62±11 n2:65±14	41/47	Acupuncture	Control	0.5–3 h before surgery	①
Wang XQ^38^	China	Craniotomy	n1:43±11 n2:41±19	40/40	TEAS	Control	30 min before induction of anesthesia lasted until 6 h after operation	②③④
Ebrahim Soltani AR^39^	Iran	Strabismus surgery	n1:19.44±10.12 n2:29.14±12.72 n3:31.17±14.79	50/100/50	Acupressure	Antiemetic; Control	30 min before induction of anesthesia	②③
Majholm^40^	Denmark	Breast Surgery	n1:62 ±2.75 n2:63 ±3.25	59/53	Acupressure	Control	Preinduction anesthesia	②
White PF^41^	America	Laparoscopic surgery	n1:46 ± 14 n2:43±11	50/50	Acupressure	Control	30 min–60 min before induction of anesthesia, maintained for 72 h	②③④
Xu M^42^	China	Craniotomy	n1:46 ± 13 n2:47 ± 11	60/59	TEAS	Control	30 min before induction of anesthesia to 24 h postoperatively	②③④
Adib-Hajbaghery M^43^	Iran	Appendectomy	n1:26.89 ±9.59 n2:31.17±14.79	35/35	Acupressure	Control	The patient was fully conscious after the operation	②③
Lee S^44^	Korea	Laparoscopic surgery	n1:46.9±13.8 n2:44.0±15.4	94/50	Acupuncture	Control	Preoperative or postoperative for 15 min	②③
Tang W^45^	China	Laparoscopic surgery	n1:39±11 n2:39±12	90/30	Electroacupuncture	Control	30 min before induction of anesthesia to the end of surgery	①
Carr KL^46^	America	Laparoscopic cholecystectomy	n1:47.00±13.40 n2:47.60±13.80	29/27	TEAS	Control	Postinduction anesthesia	①
Ertas G^47^	Turkey	Gynecologic laparoscopic surgery	n1:28.29±5.36 n2:30.19±4.75	31/31	TEAS	Control	Preoperative 15–30 min	④
Nilsson^48^	Sweden	Craniotomy	n1:56 ± 14 n2:53 ± 16	43/52	Acupressure	Control	Postoperative	①②
Yang XY^49^	China	Gynecologic laparoscopic surgery	n1:37±5.5 n2:35±9.0	50/50	TEAS+Antiemetic	Antiemetic	30 min before anesthesia induction until departure from PACU	①②③④
Praveena SS^50^	Malaysia	Total abdominal hysterectomy	n1:47.50±7.94 n2:48.72±6.72	32/32	Electroacupuncture	Control	After induction of anesthesia until the end of the operation	②
Yeoh A H^51^	Malaysia	Laparoscopic surgery	n1:41.5 ± 13.8 n2:46.5 ± 14.3	40/40	TEAS	Control	24 h after surgery	②③
Albooghobeish M^52^	Iran	Gynecological laparoscopic examination	n1:26.44±9.13 n2:25.68±8.32 n3:27.06±9.51	40/41/41	Acupuncture	Antiemetic; Control	After induction of anesthesia until the end of the operation	②③
Gilbert RT^53^	America	NA	NA	134/136	Acupressure	Control	During PACU	①
Li S^54^	China	Gynecologic laparoscopic surgery	n1:35.2±6.1 n2:34.4±9.1	20/20	Electroacupuncture+ Antiemetic	Antiemetic	Within 24 h prior to surgery for 30 min	②③
Oh H^55^	Korea	Gynecological Surgery	n1:39.78 ± 8.915 n2:43.33 ± 6.453 n3:41.17 ± 9.426	18/18/18	TEAS	Acupressure; Control	preanesthetic	④
Khoshraftar E^56^	Iran	Open abdominal surgery	NA	50/50	Acupuncture	Antiemetic	30 min before the end of surgery	①
Sahin^57^	Turkey	Laparoscopic cholecystectomy	n1:49.95±10.35 n2:48.86±10.89	37/37	Acupressure	Control	Preoperative	②③④
Unulu M^58^	Turkey	Gynecological Surgery	n1:36.4 ± 8.2 n2:39.3 ± 7.7	47/50	Acupressure	Control	Within 12 h after surgery	①
Wang TY2018^59^	China	Radical colorectal cancer surgery	n1:49.9±5.9 n2:51.4±6.3	58/20	Electroacupuncture	Control	One day preoperative, 30 min preoperative, 1 day postoperative, 30 min each time	②③
Gu SH^60^	China	Laparoscopic radical gastrectomy	n1:57.59±7.32 n2:56.67±6.23	58/59	TEAS	Control	30 min before induction of anesthesia	①
Li JL^61^	China	Laparoscopic surgery	n1:47.1±12.8 n2:47.2±10.4	105/35	TEAS	Control	30 min before induction of anesthesia to the end of surgery	①
Yang J^62^	China	Thoracoscopic surgery	n1:58.93±11.77 n2:56.39±9.34	29/28	Electroacupuncture	Control	24 h before surgery, 4 h and 24 h after surgery, 30 min each time	②③④
Li WJ^63^	China	Abdominal Surgery	n1:60±13 n2:62±12	140/140	TEAS	Control	30 min before surgery to the end of surgery, one time in the morning of the first, second, and third postoperative days, 30 min each time	①
Xiong QJ^64^	China	Laparoscopic Gastrectomy	n1:27.5±8.0 n2:27.3±8.3	31/31	TEAS+Antiemetic	Antiemetic	30 min before induction of anesthesia until the end of surgery, and two times within 12 h after surgery	①④
Gao W^65^	China	Laparoscopic non-gastrointestinal surgery	n1:39.0±7.5 n2:39.0±7.5	827/828	TEAS	Control	The day of the surgery and the next day, lasting 30 min	①
Zhu J^10^	China	Gynecologic laparoscopic surgery	n1:35.2±8.0 n2:35.7 ±8.0	299/101	Electroacupuncture	Control	one day and 30 min before surgery, 30 min each time	②③④
Honca M^66^	Turkey	Laparoscopic sleeve gastrectomy	n1:38.78±10.65 n2:37.23±10.87	30/32	Acupuncture +Antiemetic	Antiemetic	Start of anesthesia until extubation	①④
Imtiaz D^11^	Pakistan	Laparoscopic surgery for colorectal cancer	n1:42.3±4.11 n2:41.4±4.42	40/40	Acupressure	Antiemetic	30 min before to 24 h after surgery	①
Hamid^67^	Pakistan	Laparoscopic cholecystectomy	n1:38.05 ± 8.12 n2:37.56 ±8.34	43/41	Acupressure	Control	Intraoperative and 6 h postoperative	①
Yan SY^9^	China	Gynecological surgery	n1:41.9 ±12.4 n2:43.6 ±9.9	91/93	Acupuncture+Antiemetic	Antiemetic	30–60 min before surgery and during PACU, 30 min each time	①②
Qin J^8^	China	Laparoscopic gynecologic surgery	n1:45 ± 7.45 n2: 45 ± 5.92	81/81	TEAS+Antiemetic	Antiemetic	30 min before surgery	②③
Agarwal A^21^	India	Endoscopic surgery in Urology	n1:36.5±8.2 n2:34.2±6.1	100/100	Acupressure	Control	Before anesthesia begins	①
Zárate E^22^	NA	Laparoscopic cholecystectomy	n1:42± 16 n2:43±16	110/55	TEAS	Control	5–10 min before the end of the operation, wear for 9 h	②③④
Agarwal A^23^	India	Laparoscopic cholecystectomy	n1:39.2±10.4 n2:41.4±12.3 n3:40.2± 9.9	50/50/50	Acupressure	Antiemetic; Control	30 min before anesthesia induction	②③④
Coloma M^24^	America	Laparoscopic surgery	n1:42±15 n2:42±16 n3:35±9	30/30/30	TEAS+Antiemetic	TEAS; Antiemetic	NA	②③
Ming JL^25^	China	Nasal Endoscopic Surgery	NA	100/50	Acupressure	Control	1 h before surgery and 10–24 h after surgery	②③
White PF^26^	America	Plastic Operation	n1:45 ±11 n2:43 ±13 n3:46 ± 11	40/40/40	Acupressure+Antiemetic	Acupressure; Antiemetic	Wristband worn for 72 h after admission to PACU	①②③④
Samad K^27^	Pakistan	Laparoscopic cholecystectomy	n1:38.16±8.82 n2:40.60±11.11	25/25	Acupressure	Control	30 min before induction of anesthesia to 6 h postoperatively	①
Schultz AA^28^	America	Gynecological Surgery	n1:44.0±8.1 n2:47.7±12.3	24/25	Acupressure	Control	NA	②
Gan TJ^29^	America	Breast Surgery	n1:44±12 n2:47±10 n3:47±12	26/25/24	Electroacupuncture	Antiemetic; Control	At least 30 min before anesthesia until the end of surgery	③④
Klein AA^30^	America	Cardiac Surgery	n1:62±10 n2:63±8	75/77	Acupressure	Control	Worn before induction of anesthesia to 24 h after extubation	②④
Streitberger^31^	Germany	Gynecological Surgery	n1:44.3±13.47 n2:46.5±13.4	105/106	Acupuncture	Control	20 min before induction of anesthesia or after anesthesia for 20 min	②③
Amir SH^32^	India	Middle Ear Surgery	n1:17.95±8.25 n2:21.10±7.48	20/20	Electroacupuncture	Control	20 min before induction of anesthesia	①④
Sharma S^33^	India	Laparoscopic cholecystectomy	n1:47.34+6.9 n2:46.69±7.5 n3:45.41±6.4	20/20/20	Acupuncture+Antiemetic	Acupuncture; Antiemetic	5 min before anesthesia induction, lasting 30 min	②③④
liu YY^34^	China	Laparoscopic cholecystectomy	n1:42±18 n2:40±19	48/48	TEAS	Control	At least 30 min before anesthesia induction, no more than 60 min, until the end of the operation	②③④
Sadighha A^35^	Iran	Laparoscopic cholecystectomy	n1:44.4±6.6 n2:44.4±6.86 n3:45±7.32	53/51/52	Acupressure	Antiemetic; Control	NA	①②③
Korinenko Y^36^	America	Coronary artery bypass grafting and/or heart valve surgery	n1:62±11 n2:65±14	41/47	Acupuncture	Control	0.5–3 h before surgery	①
Wang XQ^37^	China	Craniotomy	n1:43±11 n2:41±19	40/40	TEAS	Control	30 min before induction of anesthesia lasted until 6 h after operation	②③④
Ebrahim Soltani AR^38^	Iran	Strabismus surgery	n1:19.44±10.12 n2:29.14±12.72 n3:31.17±14.79	50/100/50	Acupressure	Antiemetic; Control	30 min before induction of anesthesia	②③
Majholm^39^	Denmark	Breast Surgery	n1:62 ±2.75 n2:63 ±3.25	59/53	Acupressure	Control	Preinduction anesthesia	②
White PF^40^	America	Laparoscopic surgery	n1:46 ± 14 n2:43±11	50/50	Acupressure	Control	30 min–60 min before induction of anesthesia, maintained for 72 h	②③④
Xu M^41^	China	craniotomy	n1:46 ± 13 n2:47 ± 11	60/59	TEAS	Control	30 min before induction of anesthesia to 24 h postoperatively	②③④
Adib-Hajbaghery M^42^	Iran	Appendectomy	n1:26.89 ±9.59 n2:31.17±14.79	35/35	Acupressure	Control	The patient was fully conscious after the operation	②③
Lee S^43^	Korea	Laparoscopic surgery	n1:46.9±13.8 n2:44.0±15.4	94/50	Acupuncture	Control	Preoperative or postoperative for 15 min	②③
Tang W^44^	China	Laparoscopic surgery	n1:39±11 n2:39±12	90/30	Electroacupuncture	Control	30 min before induction of anesthesia to the end of surgery	①
Carr KL^45^	America	Laparoscopic cholecystectomy	n1:47.00±13.40 n2:47.60±13.80	29/27	TEAS	Control	Postinduction anesthesia	①
Ertas G^46^	Turkey	Gynecologic laparoscopic surgery	n1:28.29±5.36 n2:30.19±4.75	31/31	TEAS	Control	Preoperative 15–30 min	④
Nilsson^47^	Sweden	Craniotomy	n1:56 ± 14 n2:53 ± 16	43/52	Acupressure	Control	Postoperative	①②
Yang XY^48^	China	Gynecologic laparoscopic surgery	n1:37±5.5 n2:35±9.0	50/50	TEAS+Antiemetic	Antiemetic	30 min before anesthesia induction until departure from PACU	①②③④
Praveena SS^49^	Malaysia	Total abdominal hysterectomy	n1:47.50±7.94 n2:48.72±6.72	32/32	Electroacupuncture	Control	After induction of anesthesia until the end of the operation	②
Yeoh A H^50^	Malaysia	Laparoscopic surgery	n1:41.5 ± 13.8 n2:46.5 ± 14.3	40/40	TEAS	Control	24 h after surgery	②③
Albooghobeish M^51^	Iran	Gynecological laparoscopic examination	n1:26.44±9.13 n2:25.68±8.32 n3:27.06±9.51	40/41/41	Acupuncture	Antiemetic; Control	After induction of anesthesia until the end of the operation	②③
Gilbert RT^52^	America	NA	NA	134/136	Acupressure	Control	During PACU	①
Li S^53^	China	Gynecologic laparoscopic surgery	n1:35.2±6.1 n2:34.4±9.1	20/20	Electroacupuncture+ Antiemetic	Antiemetic	Within 24 h prior to surgery for 30 min	②③
Oh H^54^	Korea	Gynecological Surgery	n1:39.78 ± 8.915 n2:43.33 ± 6.453 n3:41.17 ± 9.426	18/18/18	TEAS	Acupressure; Control	Preanesthetic	④
Khoshraftar E^55^	Iran	Open abdominal surgery	NA	50/50	Acupuncture	Antiemetic	30 min before the end of surgery	①
Sahin^56^	Turkey	Laparoscopic cholecystectomy	n1:49.95±10.35 n2:48.86±10.89	37/37	Acupressure	Control	Preoperative	②③④
Unulu M^57^	Turkey	Gynecological Surgery	n1:36.4 ± 8.2 n2:39.3 ± 7.7	47/50	Acupressure	Control	Within 12 h after surgery	①
Wang TY2018^58^	China	Radical colorectal cancer surgery	n1:49.9±5.9 n2:51.4±6.3	58/20	Electroacupuncture	Control	One day preoperative, 30 min preoperative, 1 d postoperative, 30 min each time	②③
Gu SH^59^	China	Laparoscopic radical gastrectomy	n1:57.59±7.32 n2:56.67±6.23	58/59	TEAS	Control	30 min before induction of anesthesia	①
Li JL^60^	China	Laparoscopic surgery	n1:47.1±12.8 n2:47.2±10.4	105/35	TEAS	Control	30 min before induction of anesthesia to the end of surgery	①
Yang J^61^	China	Thoracoscopic surgery	n1:58.93±11.77 n2:56.39±9.34	29/28	Electroacupuncture	Control	24 h before surgery, 4 h and 24 h after surgery, 30 min each time	②③④
Li WJ^62^	China	Abdominal Surgery	n1:60±13 n2:62±12	140/140	TEAS	Control	30 min before surgery to the end of surgery, one time in the morning of the first, second and third postoperative days, 30 min each time	①
Xiong QJ^63^	China	Laparoscopic Gastrectomy	n1:27.5±8.0 n2:27.3±8.3	31/31	TEAS+Antiemetic	Antiemetic	30 min before induction of anesthesia until the end of surgery, and two times within 12 h after surgery	①④
Gao W^64^	China	Laparoscopic non-gastrointestinal surgery	n1:39.0±7.5 n2:39.0±7.5	827/828	TEAS	Control	The day of the surgery and the next day, lasting 30 min	①
Zhu J^10^	China	Gynecologic laparoscopic surgery	n1:35.2±8.0 n2:35.7 ±8.0	299/101	Electroacupuncture	Control	1 day and 30 min before surgery, 30 min each time	②③④
Honca M^65^	Turkey	Laparoscopic sleeve gastrectomy	n1:38.78±10.65 n2:37.23±10.87	30/32	Acupuncture +Antiemetic	Antiemetic	Start of anesthesia until extubation	①④
Imtiaz D^11^	Pakistan	Laparoscopic surgery for colorectal cancer	n1:42.3±4.11 n2:41.4±4.42	40/40	Acupressure	Antiemetic	30 min before to 24 h after surgery	①
Hamid^66^	Pakistan	Laparoscopic cholecystectomy	n1:38.05 ± 8.12 n2:37.56 ±8.34	43/41	Acupressure	Control	Intraoperative and 6 h postoperative	①
Yan SY^9^	China	Gynecological surgery	n1:41.9 ±12.4 n2:43.6 ±9.9	91/93	Acupuncture+Antiemetic	Antiemetic	30–60 min before surgery and during PACU, 30 min each time	①②
Qin J^8^	China	Laparoscopic gynecologic surgery	n1:45 ± 7.45 n2: 45 ± 5.92	81/81	TEAS+Antiemetic	Antiemetic	30 min before surgery	②③
Agarwal A^20^	India	Endoscopic surgery in Urology	n1:36.5±8.2 n2:34.2±6.1	100/100	Acupressure	Control	Before anesthesia begins	①
Zárate E^21^	NA	Laparoscopic cholecystectomy	n1:42± 16 n2:43±16	110/55	TEAS	Control	5–10 min before the end of the operation, wear for 9 h	②③④
Agarwal A^22^	India	Laparoscopic cholecystectomy	n1:39.2±10.4 n2:41.4±12.3 n3:40.2± 9.9	50/50/50	Acupressure	Antiemetic; Control	30 min before anesthesia induction	②③④
Coloma M^23^	America	Laparoscopic surgery	n1:42±15 n2:42±16 n3:35±9	30/30/30	TEAS+Antiemetic	TEAS; Antiemetic	NA	②③
Ming JL^24^	China	Nasal Endoscopic Surgery	NA	100/50	Acupressure	Control	1 h before surgery and 10–24 h after surgery	②③
White PF^26^	America	Plastic Operation	n1:45 ±11 n2:43 ±13 n3:46 ± 11	40/40/40	Acupressure+Antiemetic	Acupressure; Antiemetic	Wristband worn for 72 h after admission to PACU	①②③④
Samad K^26^	Pakistan	Laparoscopic cholecystectomy	n1:38.16±8.82 n2:40.60±11.11	25/25	Acupressure	Control	30 min before induction of anesthesia to 6 h postoperatively	①
Schultz AA^27^	America	Gynecological Surgery	n1:44.0±8.1 n2:47.7±12.3	24/25	Acupressure	Control	NA	②
Gan TJ,^28^	America	Breast Surgery	n1:44±12 n2:47±10 n3:47±12	26/25/24	Electroacupuncture	Antiemetic; Control	At least 30 min before anesthesia until the end of surgery	③④
Streitberger^30^	Germany	Gynecological Surgery	n1:44.3±13.47 n2:46.5±13.4	105/106	Acupuncture	Control	20 min before induction of anesthesia or after anesthesia for 20 min	②③
Klein AA^29^	America	Cardiac Surgery	n1:62±10 n2:63±8	75/77	Acupressure	Control	Worn before induction of anesthesia to 24 h after extubation	②④
Amir SH^31^	India	Middle Ear Surgery	n1:17.95±8.25 n2:21.10±7.48	20/20	Electroacupuncture	Control	20 min before induction of anesthesia	①④
Sharma S^32^	India	Laparoscopic cholecystectomy	n1:47.34+6.9 n2:46.69±7.5 n3:45.41±6.4	20/20/20	Acupuncture+Antiemetic	Acupuncture; Antiemetic	5 min before anesthesia induction, lasting 30 min	②③④
liu YY^33^	China	Laparoscopic cholecystectomy	n1:42±18 n2:40±19	48/48	TEAS	Control	At least 30 min before anesthesia induction, no more than 60 min, until the end of the operation	②③④
Sadighha A^34^	Iran	Laparoscopic cholecystectomy	n1:44.4±6.6 n2:44.4±6.86 n3:45±7.32	53/51/52	Acupressure	Antiemetic; Control	NA	①②③
Korinenko Y^35^	America	Coronary artery bypass grafting and/or heart valve surgery	n1:62±11 n2:65±14	41/47	Acupuncture	Control	0.5–3 h before surgery	①
Wang XQ^36^	China	Craniotomy	n1:43±11 n2:41±19	40/40	TEAS	Control	30 min before induction of anesthesia lasted until 6 h after operation	②③④
Majholm^38^	Denmark	Breast Surgery	n1:62 ±2.75 n2:63 ±3.25	59/53	Acupressure	Control	Preinduction anesthesia	②
Ebrahim Soltani AR^37^	Iran	Strabismus surgery	n1:19.44±10.12 n2:29.14±12.72 n3:31.17±14.79	50/100/50	Acupressure	Antiemetic; Control	30 min before induction of anesthesia	②③
Xu M^40^	China	Craniotomy	n1:46 ± 13 n2:47 ± 11	60/59	TEAS	Control	30 min before induction of anesthesia to 24 h postoperatively	②③④
White PF^39^	America	Laparoscopic surgery	n1:46 ± 14 n2:43±11	50/50	Acupressure	Control	30 min–60 min before induction of anesthesia, maintained for 72 h	②③④
Lee S^42^	Korea	Laparoscopic surgery	n1:46.9±13.8 n2:44.0±15.4	94/50	Acupuncture	Control	Preoperative or postoperative for 15 min	②③
Tang W^43^	China	Laparoscopic surgery	n1:39±11 n2:39±12	90/30	Electroacupuncture	Control	30 min before induction of anesthesia to the end of surgery	①
Adib-Hajbaghery M^41^	Iran	Appendectomy	n1:26.89 ±9.59 n2:31.17±14.79	35/35	Acupressure	Control	The patient was fully conscious after the operation	②③
Carr KL^44^	America	Laparoscopic cholecystectomy	n1:47.00±13.40 n2:47.60±13.80	29/27	TEAS	Control	Postinduction anesthesia	①
Ertas G^45^	Turkey	Gynecologic laparoscopic surgery	n1:28.29±5.36 n2:30.19±4.75	31/31	TEAS	Control	Preoperative 15–30 min	④
Yang XY^47^	China	Gynecologic laparoscopic surgery	n1:37±5.5 n2:35±9.0	50/50	TEAS+Antiemetic	Antiemetic	30 min before anesthesia induction until departure from PACU	①②③④
Nilsson^46^	Sweden	Craniotomy	n1:56 ± 14 n2:53 ± 16	43/52	Acupressure	Control	Postoperative	①②
Yeoh A H^49^	Malaysia	Laparoscopic surgery	n1:41.5 ± 13.8 n2:46.5 ± 14.3	40/40	TEAS	Control	24 h after surgery	②③
Praveena SS^48^	Malaysia	Total abdominal hysterectomy	n1:47.50±7.94 n2:48.72±6.72	32/32	Electroacupuncture	Control	After induction of anesthesia until the end of the operation	②
Li S^53^	China	Gynecologic laparoscopic surgery	n1:35.2±6.1 n2:34.4±9.1	20/20	Electroacupuncture+ Antiemetic	Antiemetic	Within 24 h prior to surgery for 30 min	②③
Albooghobeish M^50^	Iran	Gynecological laparoscopic examination	n1:26.44±9.13 n2:25.68±8.32 n3:27.06±9.51	40/41/41	Acupuncture	Antiemetic; Control	After induction of anesthesia until the end of the operation	②③
Oh H^54^	Korea	Gynecological Surgery	n1:39.78 ± 8.915 n2:43.33 ± 6.453 n3:41.17 ± 9.426	18/18/18	TEAS	Acupressure; Control	Preanesthetic	④
Gilbert RT^51^	America	NA	NA	134/136	Acupressure	Control	During PACU	①
Khoshraftar E^55^	Iran	Open abdominal surgery	NA	50/50	Acupuncture	Antiemetic	30 min before the end of surgery	①
Sahin^56^	Turkey	Laparoscopic cholecystectomy	n1:49.95±10.35 n2:48.86±10.89	37/37	Acupressure	Control	Preoperative	②③④
Unulu M^57^	Turkey	Gynecological Surgery	n1:36.4 ± 8.2 n2:39.3 ± 7.7	47/50	Acupressure	Control	Within 12 h after surgery	①
Wang TY^58^	China	Radical colorectal cancer surgery	n1:49.9±5.9 n2:51.4±6.3	58/20	Electroacupuncture	Control	One day preoperative, 30 min preoperative, 1 day postoperative, 30 min each time	②③
Gu SH^59^	China	Laparoscopic radical gastrectomy	n1:57.59±7.32 n2:56.67±6.23	58/59	TEAS	Control	30 min before induction of anesthesia	①
Li JL^60^	China	Laparoscopic surgery	n1:47.1±12.8 n2:47.2±10.4	105/35	TEAS	Control	30 min before induction of anesthesia to the end of surgery	①
Yang J^61^	China	Thoracoscopic surgery	n1:58.93±11.77 n2:56.39±9.34	29/28	Electroacupuncture	Control	24 h before surgery, 4 h and 24 h after surgery, 30 min each time	②③④
Li WJ^62^	China	Abdominal Surgery	n1:60±13 n2:62±12	140/140	TEAS	Control	30 min before surgery to the end of surgery, one time in the morning of the first, second and third postoperative days, 30 min each time	①
Xiong QJ^63^	China	Laparoscopic Gastrectomy	n1:27.5±8.0 n2:27.3±8.3	31/31	TEAS+Antiemetic	Antiemetic	30 min before induction of anesthesia until the end of surgery, and two times within 12 h after surgery	①④
Gao W^64^	China	Laparoscopic non-gastrointestinal surgery	n1:39.0±7.5 n2:39.0±7.5	827/828	TEAS	Control	The day of the surgery and the next day, lasting 30 min	①
Zhu J^10^	China	Gynecologic laparoscopic surgery	n1:35.2±8.0 n2:35.7 ±8.0	299/101	Electroacupuncture	Control	One day and 30 min before surgery, 30 min each time	②③④
Honca M^65^	Turkey	Laparoscopic sleeve gastrectomy	n1:38.78±10.65 n2:37.23±10.87	30/32	Acupuncture +Antiemetic	Antiemetic	Start of anesthesia until extubation	①④
Imtiaz D^11^	Pakistan	Laparoscopic surgery for colorectal cancer	n1:42.3±4.11 n2:41.4±4.42	40/40	Acupressure	Antiemetic	30 min before to 24 h after surgery	①
Hamid^66^	Pakistan	Laparoscopic cholecystectomy	n1:38.05 ± 8.12 n2:37.56 ±8.34	43/41	Acupressure	Control	Intraoperative and 6 h postoperative	①
Yan SY^9^	China	Gynecological surgery	n1:41.9 ±12.4 n2:43.6 ±9.9	91/93	Acupuncture+Antiemetic	Antiemetic	30–60 min before surgery and during PACU, 30 min each time	①②
Qin J^8^	China	Laparoscopic gynecologic surgery	n1:45 ± 7.45 n2: 45 ± 5.92	81/81	TEAS+Antiemetic	Antiemetic	30 min before surgery	②③

Note: ①: incidence of postoperative nausea and vomiting; ②: incidence of postoperative nausea; ③: incidence of postoperative vomiting; ④: number of patients need antiemetic rescue.

### Risk of bias assessment

**Figure 2:**
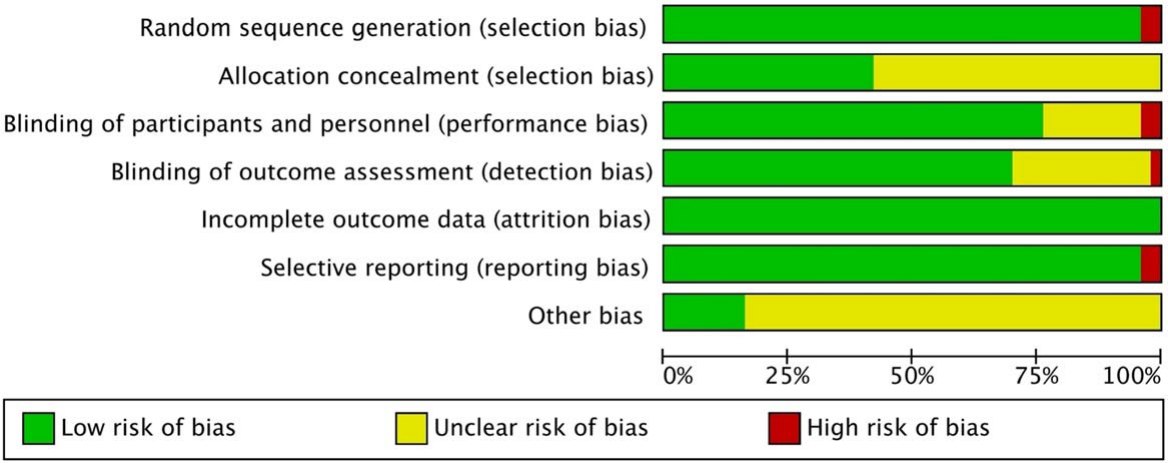
Risk bias assessment graph for the included studies.

Of the 50 included RCTs, 48 studies were low risk for randomised sequence generation and only two were high-risk. Twenty-eight RCTs did not describe detailed allocation concealment methods, 2 RCTs had a high-risk of blinding of participants and personnel, and a further 38 RCTs explicitly mentioned the implementation of double blinding of subjects and personnel. Almost all RCTs were judged to be at low risk of bias for incomplete outcome data. Two RCTs were at high-risk for selective reporting, while the remaining 47 RCTs were at low risk of bias. The risk of bias assessment for the included RCTS is shown in Figure 2 and Figure S1 (Supplemental Digital Content 4, http://links.lww.com/JS9/D442).

### Incidence of PONV

**Figure 3:**
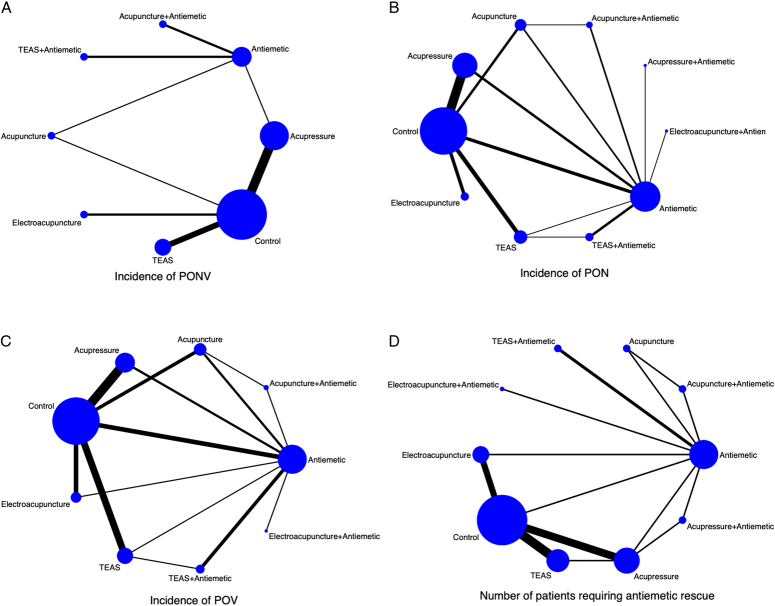
The network plots. A, incidence of postoperative nausea and vomiting; B, incidence of postoperative nausea; C, incidence of postoperative vomiting; D, number of patients need antiemetic rescue.

**Figure 4:**
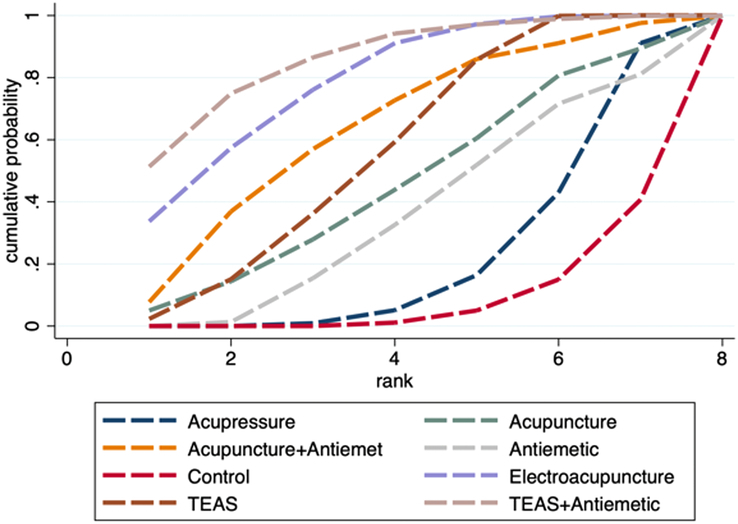
SUCRA ranking curve of PONV incidence.

A total of 22 studies involving 4103 participants were included to compare the effect of acupoint stimulation on the incidence of PONV in patients undergoing general anesthesia. The network plot is shown in Figure 3A. Both the global and local inconsistency tests suggested that there was no significant inconsistency between direct and indirect comparisons (*P*=0.496) (Fig. S2, Supplemental Digital Content 4, http://links.lww.com/JS9/D442 and Table S1, Supplemental Digital Content 4, http://links.lww.com/JS9/D442), which demonstrated that a consistency model should be used. The results of the network meta-analysis showed that compared with control (sham acupoint stimulation or blank control), antiemetic alone did not significantly reduce the incidence of PONV (RR 0.71, 95% CI: 0.29–1.73); however, antiemetic combined with TEAS presented significant reduction (RR 0.53, 95% CI: 0.34–0.82); meanwhile, both electroacupuncture alone and TEAS alone could significantly reduce the incidence of PONV (RR 0.43, 95% CI: 0.26–0.72; RR 0.60, 95% CI: 0.46–0.77; respectively), and they were more effective than acupressure in reducing PONV (RR 0.49, 95% CI: 0.28–0.84; RR 0.67, 95% CI: 0.49–0.92; respectively) (Table 2). The SUCRA probability ranking results revealed that TEAS combined with antiemetic showed the biggest possibility to be the best intervention, followed by electroacupuncture, acupuncture combined with antiemetic, TEAS, acupuncture, antiemetic, acupressure, and control (Fig. 4 and Table S2, Supplemental Digital Content 4, http://links.lww.com/JS9/D442).

**Table 2:** Results of network meta-analysis of the incidence of PONV [RR (95% CI)].

TEAS+Antiemetic							
0.86 (0.28–2.64)	Electroacupuncture						
0.72 (0.38–1.36)	0.83 (0.27–2.55)	Acupuncture+Antiemet					
0.63 (0.23–1.75)	0.73 (0.41–1.28)	0.87 (0.31–2.43)	TEAS				
0.56 (0.17–1.77)	0.64 (0.27–1.52)	0.77 (0.24–2.46)	0.89 (0.42–1.85)	Acupuncture			
**0.53 (0.34–0.82)**	0.61 (0.22–1.70)	0.73 (0.47–1.15)	0.84 (0.33–2.11)	0.95 (0.33–2.76)	Antiemetic		
0.42 (0.16–1.12)	**0.49 (0.28**–**0.84)**	0.58 (0.22–1.55)	**0.67 (0.49**–**0.92)**	0.75 (0.37–1.54)	0.79 (0.33–1.90)	Acupressure	
0.38 (0.14–1.02)	**0.43 (0.26**–**0.72)**	0.52 (0.19–1.41)	**0.60 (0.46**–**0.77)**	0.67 (0.34–1.34)	0.71 (0.29–1.73)	0.89 (0.73–1.10)	Control

Bold values are statistically significant.

### Incidence of PON

**Figure 5:**
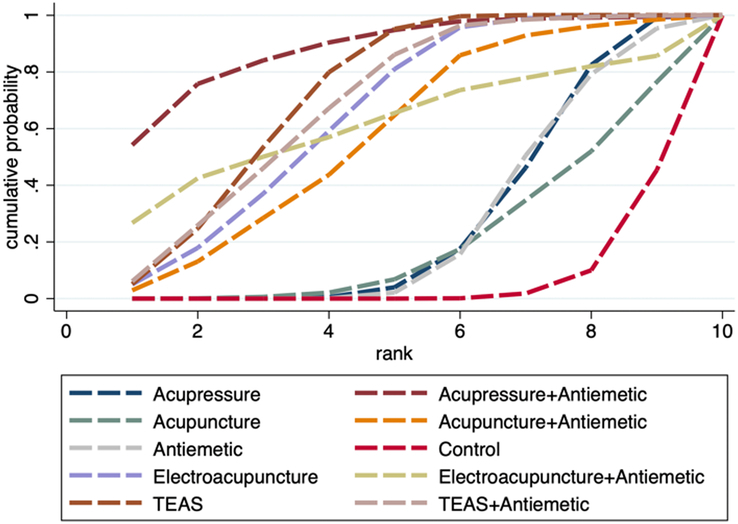
SUCRA ranking curve of PON incidence.

Thirty RCTs involving 3647 participants reported the incidence of PON, and the network diagram is shown in Figure 3B. Neither the global nor the local inconsistency tests found significant inconsistency, so the consistency model was used (*P*=0.444 ) (Fig. S5, Supplemental Digital Content 4, http://links.lww.com/JS9/D442 and Table S3, Supplemental Digital Content 4, http://links.lww.com/JS9/D442). According to the network meta-analysis results, compared to the control (sham acupoint stimulation or blank control), neither acupuncture alone nor acupuncture combined with antiemetic significantly decreased the incidence of PON (RR 0.87, 95% CI: 0.56–1.36; RR 0.56, 95% CI: 0.31–1.02; respectively); nevertheless, acupressure alone and acupressure combined with antiemetic significantly decreased the incidence (RR 0.81, 95% CI: 0.68–0.98; RR 0.32, 95% CI: 0.14–0.77, respectively); in the meantime, both TEAS alone and TEAS in combination with antiemetic might considerably lower the incidence of PON (RR 0.48, 95% CI: 0.36–0.63; and RR 0.49, 95% CI: 0.29–0.83, respectively) (Table 3). Acupressure combined with antiemetic was found to have the highest likelihood of being the best intervention according to the SUCRA probability ranking results, preceded by TEAS, TEAS combined with antiemetic, electroacupuncture, electroacupuncture combined with antiemetic, acupuncture combined with antiemetic, antiemetic alone, acupuncture alone, and control (Fig. 5 and Table 4).

**Table 3:** Results of network meta-analysis of the incidence of PON [RR (95% CI)].

Acupressure+Antiemetic									
0.67 (0.28–1.64)	TEAS								
0.65 (0.26–1.66)	0.97 (0.57–1.67)	TEAS+Antiemetic							
0.62 (0.24–1.59)	0.92 (0.59–1.45)	0.95 (0.51–1.79)	electroacupuncture						
0.67 (0.14–3.24)	0.99 (0.24–4.04)	1.02 (0.24–4.26)	1.07 (0.25–4.51)	electroacupuncture+Antiemetic					
0.57 (0.22–1.48)	0.85 (0.45–1.58)	0.87 (0.44–1.72)	0.92 (0.46–1.83)	0.86 (0.20–3.64)	Acupuncture+Antiemetic				
**0.40 (0.17**–**0.94)**	**0.59 (0.43**–**0.81)**	**0.61 (0.39**–**0.96)**	0.64 (0.40–1.03)	0.59 (0.15–2.39)	0.69 (0.38–1.25)	Acupressure			
**0.40 (0.18**–**0.90)**	**0.59 (0.41**–**0.86)**	**0.61 (0.39**–**0.96)**	0.64 (0.40–1.03)	0.60 (0.15–2.33)	0.70 (0.42–1.16)	1.01 (0.74–1.39)	Antiemetic		
**0.37 (0.14**–**0.97)**	**0.55 (0.33**–**0.92)**	0.56 (0.29–1.10)	0.59 (0.34–1.05)	0.55 (0.13–2.37)	0.65 (0.32–1.33)	0.93 (0.58–1.49)	0.92 (0.55–1.55)	Acupuncture	
**0.32 (0.14**–**0.77)**	**0.48 (0.36**–**0.63)**	**0.49 (0.29**–**0.83)**	**0.52 (0.36**–**0.74)**	0.48 (0.12–1.94)	0.56 (0.31–1.02)	**0.81 (0.68**–**0.98)**	0.81 (0.59–1.10)	0.87 (0.56–1.36)	Control

Bold values are statistically significant.

**Table 4:** Results of network meta-analysis of the incidence of POV [RR (95% CI)].

Electroacupuncture+Antiemetic								
0.64 (0.02–22.93)	Acupuncture+Antiemet							
0.24 (0.03–1.91)	0.37 (0.02–7.31)	Acupressure						
0.20 (0.03–1.63)	0.32 (0.02–6.25)	0.85 (0.53–1.38)	Electroacupuncture					
0.21 (0.02–1.72)	0.32 (0.02–6.54)	0.87 (0.42–1.78)	1.02 (0.50–2.08)	TEAS+Antiemetic				
0.17 (0.02–1.28)	0.26 (0.01–5.01)	0.70 (0.45–1.10)	0.82 (0.53–1.29)	0.81 (0.45–1.47)	Antiemetic			
0.15 (0.02–1.21)	0.23 (0.01–4.55)	0.62 (0.36–1.05)	0.72 (0.41–1.28)	0.71 (0.32–1.56)	0.87 (0.50–1.53)	Acupuncture		
0.15 (0.02–1.18)	0.23 (0.01–4.54)	**0.62 (0.39**–**0.98)**	0.73 (0.45–1.17)	0.72 (0.37–1.40)	0.88 (0.57–1.35)	1.01 (0.59–1.74)	TEAS	
**0.08 (0.01**–**0.65)**	0.13 (0.01–2.51)	**0.35 (0.25**–**0.48)**	**0.41 (0.29**–**0.58)**	**0.40 (0.21**–**0.77)**	**0.49 (0.36**–**0.69)**	**0.57 (0.37**–**0.87)**	**0.56 (0.40**–**0.78)**	Control

Bold values are statistically significant.

### Incidence of POV

**Figure 6:**
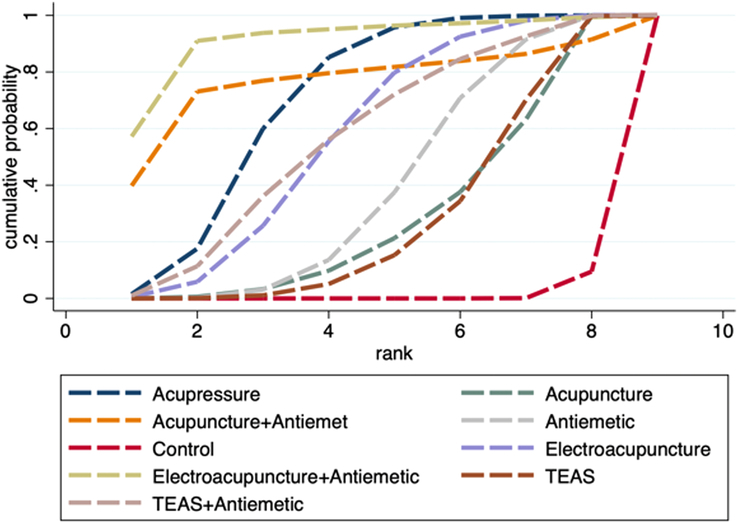
SUCRA ranking curve of POV incidence.

Twenty-six RCTs involving 3080 participants reported the incidence of POV. The network relationships are plotted in Figure 3C. Both global and local inconsistency tests did not reveal significant inconsistencies (*P*=0.204), and so the consistency model was selected (Fig. S8, Supplemental Digital Content 4, http://links.lww.com/JS9/D442 and Table S5, Supplemental Digital Content 4, http://links.lww.com/JS9/D442). The results of the network meta-analysis showed that compared with control (sham acupoint stimulation or blank control), antiemetic alone significantly reduced the incidence of POV (RR 0.49, 95% CI: 0.36–0.69); concurrently, the incidence of POV was significantly lower when antiemetics were combined with TEAS or electroacupuncture (RR 0.40, 95% CI: 0.21–0.77; RR 0.08, 95% CI: 0.01–0.65; respectively); however, acupressure was more effective in preventing POV compared to TEAS (RR 0.62, 95% CI: 0.39–0.98) (Table 4). The SUCRA probability ranking results revealed that electroacupuncture combined with antiemetic had the highest likelihood of being the most effective intervention, followed by acupuncture combined with antiemetic, acupressure, electroacupuncture, TEAS combined with antiemetic, antiemetic alone, acupuncture alone, TEAS alone, and control (Fig. 6 and Table S6, Supplemental Digital Content 4, http://links.lww.com/JS9/D442).

### Number of patients needing antiemetic rescue

**Figure 7:**
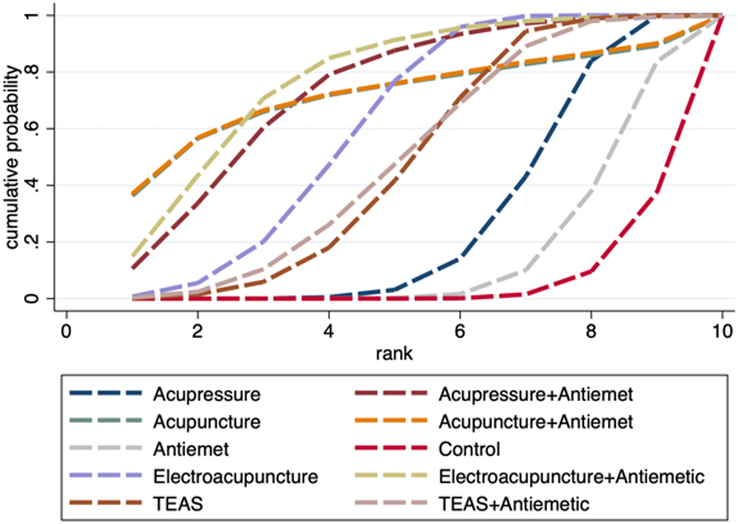
SUCRA ranking curve of number of patients needing antiemetic rescue.

Nineteen studies with 1978 participants reported the number of patients who required rescue antiemetic. See Figure 3D for the network diagram. There was no significant inconsistency between direct and indirect comparisons according to both global and local inconsistency tests (*P*=0.088), indicating the need for a consistency model (Fig. S11, Supplemental Digital Content 4, http://links.lww.com/JS9/D442 and Table S7, Supplemental Digital Content 4, http://links.lww.com/JS9/D442). The results of the network meta-analysis showed that compared with control (sham acupoint stimulation or blank control), antiemetic alone did not significantly reduce the number of patients requiring rescue antiemetic (RR 0.81, 95% CI: 0.48–1.40); nevertheless, antiemetic in combination with electroacupuncture, acupressure, and TEAS, respectively, showed significant reduction (RR 0.22, 95% CI: 0.07–0.68; RR 0.25, 95% CI: 0.09–0.73; RR 0.45, 95% CI: 0.21–0.96; respectively); meanwhile, compare with antiemetic, the number of patients who needed rescue antiemetic drugs was lower in electroacupuncture (RR 0.46, 95% CI: 0.26–0.83) (Table 5). The SUCRA probability ranking results revealed that electroacupuncture combined with antiemetic showed the biggest possibility to be the best intervention, followed by acupressure combined with antiemetic, acupuncture combined with antiemetic, acupuncture, electroacupuncture, TEAS combined with antiemetic, TEAS, acupressure, antiemetic, control (Fig. 7 and Table S8, Supplemental Digital Content 4, http://links.lww.com/JS9/D442).

**Table 5:** Results of network meta-analysis of number of patients needing antiemetic rescue [RR (95% CI).

Electroacupuncture+Antiemetic									
0.87 (0.21–3.58)	Acupressure+Antiemet								
1.34 (0.06–30.85)	1.53 (0.07–35.42)	Acupuncture+Antiemet							
1.34 (0.06–30.85)	1.53 (0.07–35.42)	1.00 (0.02–48.09)	Acupuncture						
0.58 (0.18–1.83)	0.66 (0.22–1.99)	0.43 (0.02–8.94)	0.43 (0.02–8.94)	Electroacupuncture					
0.49 (0.16–1.52)	0.56 (0.18–1.75)	0.37 (0.02–7.52)	0.37 (0.02–7.52)	0.85 (0.38–1.88)	TEAS+Antiemetic				
0.47 (0.15–1.54)	0.54 (0.18–1.67)	0.35 (0.02–7.40)	0.35 (0.02–7.40)	0.82 (0.54–1.25)	0.97 (0.42–2.23)	TEAS			
0.34 (0.11–1.04)	0.38 (0.13–1.12)	0.25 (0.01–5.16)	0.25 (0.01–5.16)	0.58 (0.41–0.83)	0.69 (0.32–1.48)	0.71 (0.47–1.08)	Acupressure		
**0.27 (0.10**–**0.72)**	**0.31 (0.11**–**0.84)**	0.20 (0.01–3.92)	0.20 (0.01–3.92)	**0.46 (0.26–0.83)**	**0.55 (0.32–0.94)**	0.57 (0.30–1.07)	0.80 (0.46–1.38)	Antiemetic	
**0.22 (0.07**–**0.68)**	**0.25 (0.09**–**0.73)**	0.16 (0.01–3.35)	0.16 (0.01–3.35)	**0.38 (0.29–0.49)**	**0.45 (0.21–0.96)**	**0.46 (0.33–0.65)**	**0.65 (0.51–0.83)**	0.81 (0.48–1.40)	Control

Bold values are statistically significant.

## Discussion

### Principal findings

This systematic review and network meta-analysis included 50 RCTs that compared the efficacy of different acupoint stimulation techniques to prevent PONV. We found that antiemetics alone did not significantly reduce the incidence of PONV and PON; whereas TEAS combined with antiemetics significantly reduced the incidence of PONV, PON, and POV simultaneously and was the optimal measure to reduce the incidence of PONV; electroacupuncture combined with antiemetics was effective in preventing the incidence of POV, thus reducing the number of patients requiring rescue antiemetics; and compared with other interventions, acupressure combined with antiemetics was the best intervention to reduce PON. Overall, electrical stimulation was more effective than physical stimulation in preventing postoperative nausea and/or vomiting, and combination with antiemetics was the most effective strategy.

### Advantages and limitations

In our research, we conducted a thorough investigation that involved a comprehensive synthesis of evidence on the effectiveness of acupoint stimulation in preventing postoperative nausea and/or vomiting. We systematically reviewed a large number of clinical studies and trials and evaluated their efficacy in the treatment of PONV to draw reliable conclusions. Our findings suggest that acupoint stimulation may be a potential treatment for the prevention and control of PONV. However, the following limitations still exist in this study: first, the included studies were mainly from Asian countries, especially in China, South Korea, and several Southeast Asian countries, which may limit the application of the results in other areas; second, in the risk of bias assessment of the literature, the limited literature did not detail the allocation of randomized sequence generation and blinded conduct, and some of the literature had a high-risk of bias, which may reduce the reliability of the results; lastly, as 13 of the studies included 1668 patients undergoing gynecological or breast surgeries, and females are one of the risk factors for PONV, there is a higher proportion of females among the included patients. It is currently unclear whether the higher proportion of females would affect the analysis results.

### Comparison with other studies

A recently published study on TEAS for the prevention of PONV demonstrated the effectiveness of TEAS in reducing the incidence of PON, POV, and PONV^17^. According to the 2020 General Guidelines for the Management of Postoperative Nausea and Vomiting (Fourth Edition), risk factors for PONV include female sex, history of PONV/motion sickness, younger age, nonsmoker, opioid analgesia, and surgery type. Low risk refers to no risk factors; medium risk includes 1–2 risk factors; high-risk consists of three or more risk factors^1^. And the addition of TEAS therapy combined with palonosetron or dexamethasone is recommended for high-risk PONV patients^1^. Furthermore, TEAS has been demonstrated to lessen pain, lessen anxiety, prevent preinduction hypertension, and improve the quality of early recovery^68–70^. Low postoperative opioid intake is associated with lower pain scores, which further lowers the risk of PONV. Our analysis came to the same conclusion: the best treatment for preventing nausea and vomiting following general anesthesia is TEAS in combination with antiemetics.

Previous studies have shown that acupressure could help in reducing the incidence and severity of nausea, but not vomiting or dry heaving^71,72^. However, our results indicated that acupressure not only reduced the incidence of nausea but also helped in reducing the incidence of vomiting. The mechanism behind this was the increased release of β-endorphin into the cerebrospinal fluid by the hypothalamus, which had a clear neurobiological basis as β-endorphin was believed to have antiemetic effects^73^.

## Conclusion

Our NMA study showed that electrical stimulation of acupoints, including electroacupuncture and TEAS, reduced the incidence of postoperative nausea and vomiting after general anesthesia, postoperative nausea, and postoperative vomiting, and reduced the number of patients needing antiemetic rescue. Although limited by the quality and sample size of the included studies, the results of this study may be helpful in developing guidelines for acupoint stimulation for the prevention of postoperative nausea and vomiting.

## Ethical approval

Not applicable.

## Consent

Not applicable.

## Source of funding

The study was supported by the evidence-based research and formulation of clinical practice guidelines for the prevention and treatment of postoperative gastrointestinal dysfunction by integrating Chinese and Western medicine (2023-2-53) and formulation of clinical practice guidelines and development of perioperative clinical decision-making system for prevention and treatment of postoperative gastrointestinal dysfunction by integrating Chinese and Western medicine (22YF7FA101).

## Author contribution

T.Z., H.H., and Y.W.: study conception and design; C.H.W., R.L., P.W., and M.W.: acquisition of data; D.L. and F.T.: analysis and interpretation of data; T.Z., H.H., Z.C.: drafting of manuscript; L.G., J.X., and S.Z.: critical revision.

## Conflicts of interest disclosure

The authors declare no conflicts of interest.

## Research registration unique identifying number (UIN)

1. Name of the registry: PROSPERO.
2. Unique identifying number or registration ID: CRD42023446856.
3. Hyperlink to your specific registration (must be publicly accessible and will be checked): https://www.crd.york.ac.uk/prospero/#myprospero.

## Guarantor

Jianjun Xue, Prof. Anesthesia and Pain Medical Center, Gansu Hospital of Traditional Chinese Medicine, Lanzhou, Gansu 730050, People’s Republic of China. E-mail: xjjfei419@126.com.

## Data availability statement

The data that support the findings of this study are available from the corresponding author upon reasonable request.

## Provenance and peer review

Not commissioned, externally peer-reviewed.

## Supplementary Material
