# Supplementary material for: Comparative effectiveness of acupoint stimulation for preventing postoperative nausea and vomiting after general anesthesia: a network meta-analysis of randomized trials
Source: Int J Surg. 2024 Sep 19;111(1):1330–47. doi: 10.1097/JS9.0000000000001976 (PMC11745642; doi:10.1097/JS9.0000000000001976)
Supplement: Supplementary file 2 [file js9-111-1330-s002.doc]

Identification

Records identified through database searching(n=10662)

Additional records identified through other sources(n=0)

Screening

Eligibility

Included

Records after removing duplicates(n=4538)

Excluded apparently irrelevant records(n=4370)

Excluded (n=118)

1. Duplicate publications (n=12)

2. Ending indicators do not match (n=68)

3.Data not available (n=13)

4. Protocol (n=25)

Full-text article reviewed for eligibility(n=168)

Studies included in this meta-anaiysis (n=50)

Records screened through title and abstract(n=4538)

Fig. 1. Flow diagram of study selection
