# Supplementary material for: Comparative effectiveness of acupoint stimulation for preventing postoperative nausea and vomiting after general anesthesia: a network meta-analysis of randomized trials
Source: Int J Surg. 2024 Sep 19;111(1):1330–47. doi: 10.1097/JS9.0000000000001976 (PMC11745642; doi:10.1097/JS9.0000000000001976)
Supplement: Supplementary file 4 [file js9-111-1330-s004.docx]

**Figure S1：Risk of bias summary: review authors' judgements about each risk of bias item for each included study**


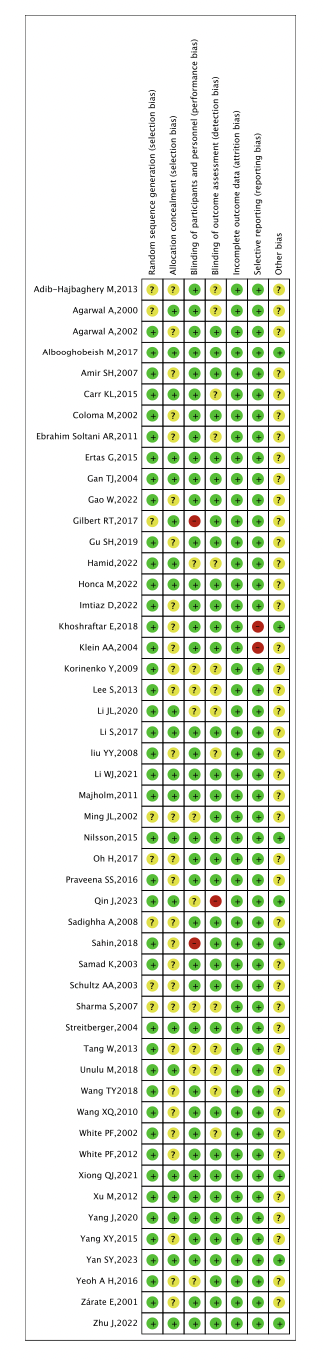


**Figure S2: Forest plot and the global inconsistency test result (Incidence of PONV)**

(chi2=0.46, p=0.496)


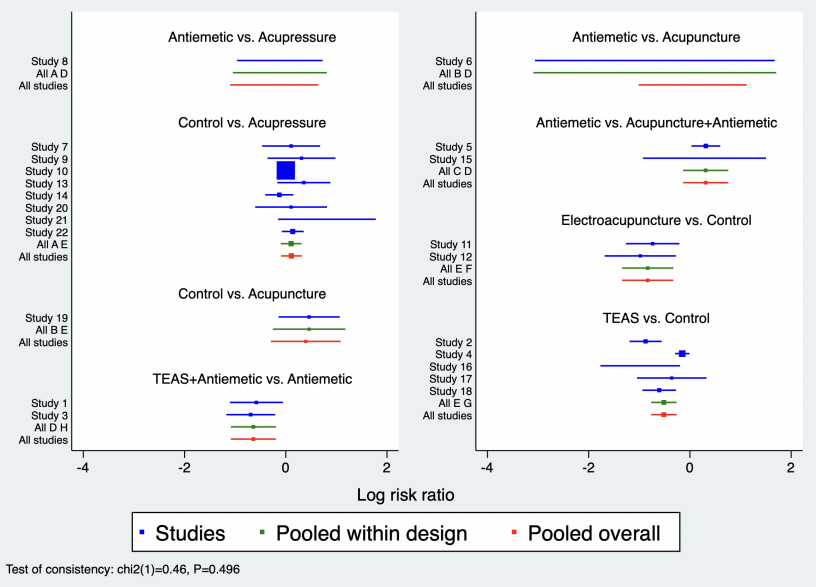


Label: A : Acupressure B: Acupuncture C: Acupuncture+Antiemetic D: Antiemetic E: Control F: Electroacupuncture G: TEAS H: TEAS+Antiemetic

**Table S1: Local inconsistency test (Incidence of PONV)**


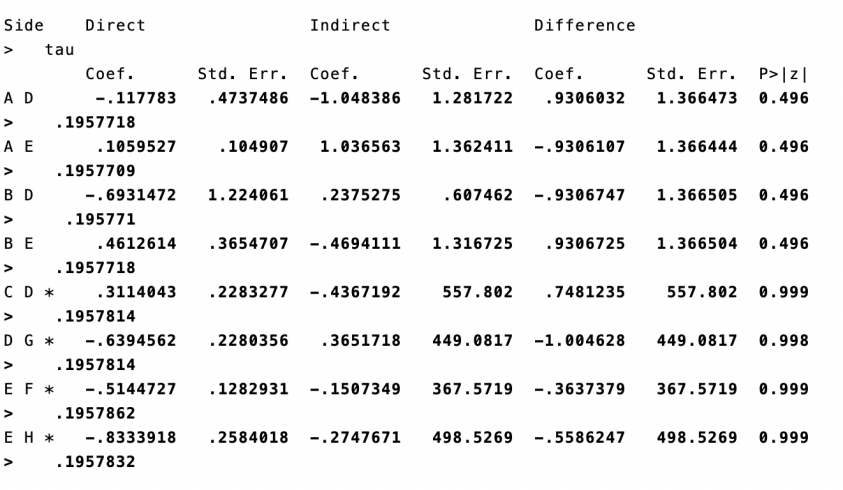


Label: A : Acupressure B: Acupuncture C: Acupuncture+Antiemetic D: Antiemetic E: Control F: Electroacupuncture G: TEAS H: TEAS+Antiemetic

**Table S2: SUCRA analysis: MeanRank table (Incidence of PONV)**


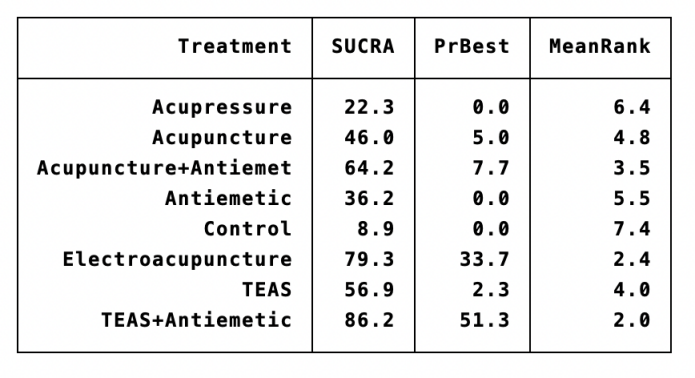


**Figure S3: SUCRA analysis: MeanRank figure (Incidence of PONV)**


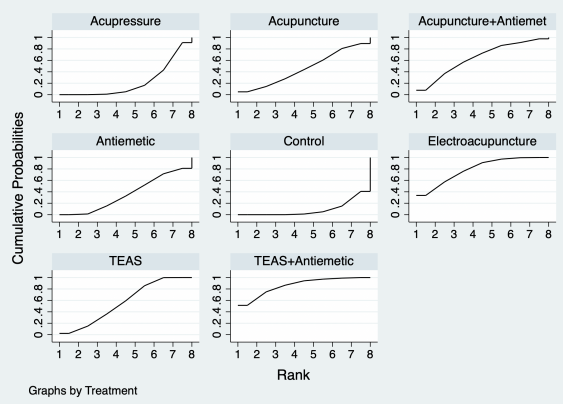


**Figure S4: Funnel diagram(Incidence of PONV)**


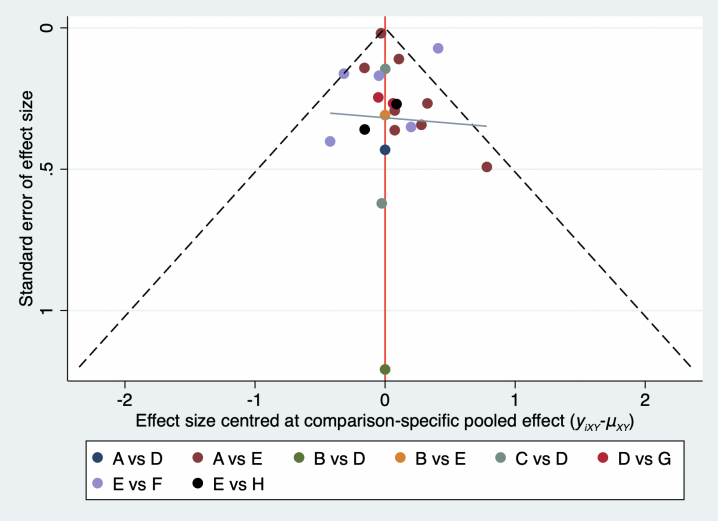


Label: A : Acupressure B: Acupuncture C: Acupuncture+Antiemetic D: Antiemetic E: Control F: Electroacupuncture G: TEAS H: TEAS+Antiemetic

**Figure S5: Forest plot and the global inconsistency test result (Incidence of PON)**

(chi2=6.86, p=0.444)


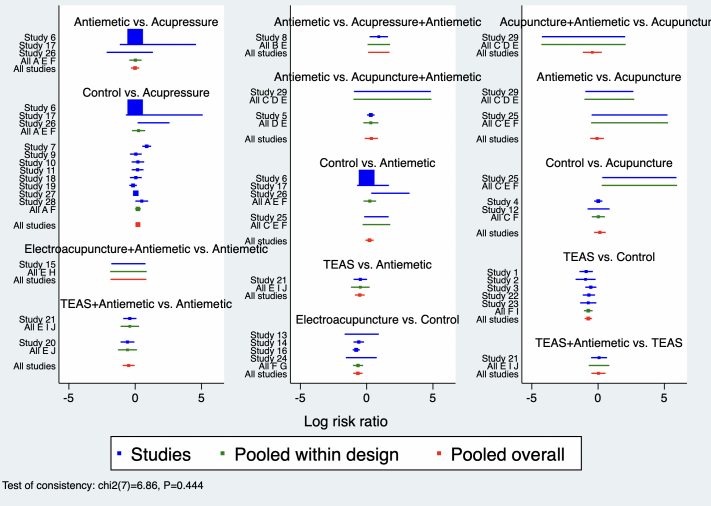


Label: A: Acupressure B: Acupressure+Antiemetic C: Acupuncture D: Acupuncture+Antiemetic E: Antiemetic F: Control G: Electroacupuncture H: Electroacupuncture+Antiemetic I: TEAS J: TEAS+Antiemetic

**Table S3: Local inconsistency test (Incidence of PON)**


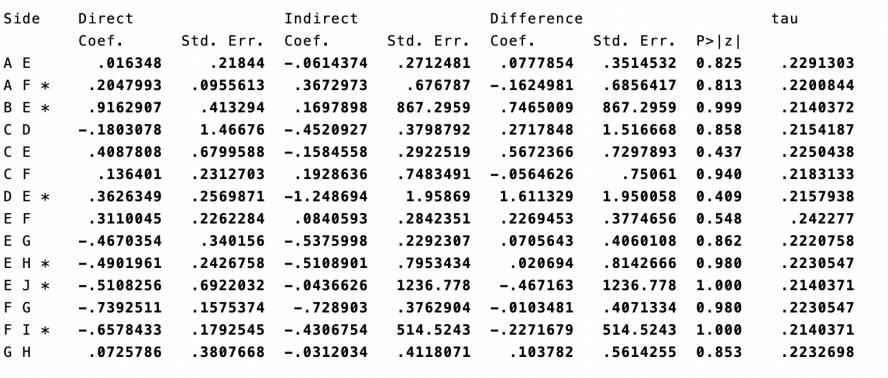


Label: A: Acupressure B: Acupressure+Antiemetic C: Acupuncture D: Acupuncture+Antiemetic E: Antiemetic F: Control G: Electroacupuncture H: Electroacupuncture+Antiemetic I: TEAS J: TEAS+Antiemetic

**Table S4: SUCRA analysis: MeanRank table (Incidence of PON)**


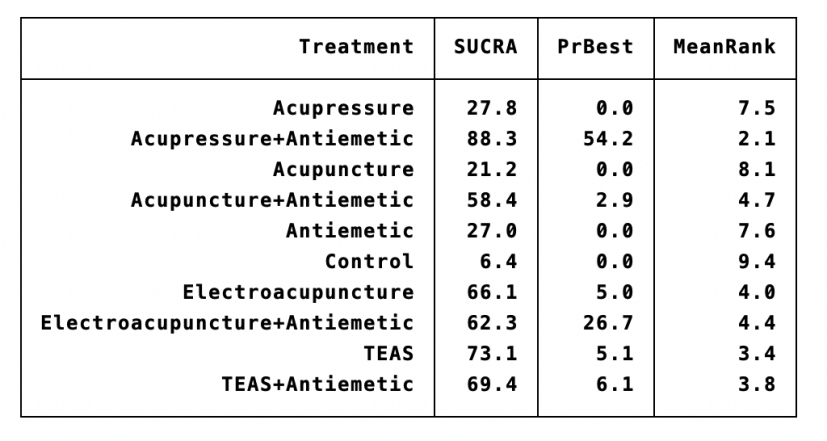


**Figure S6: SUCRA analysis: MeanRank figure (Incidence of PON)**


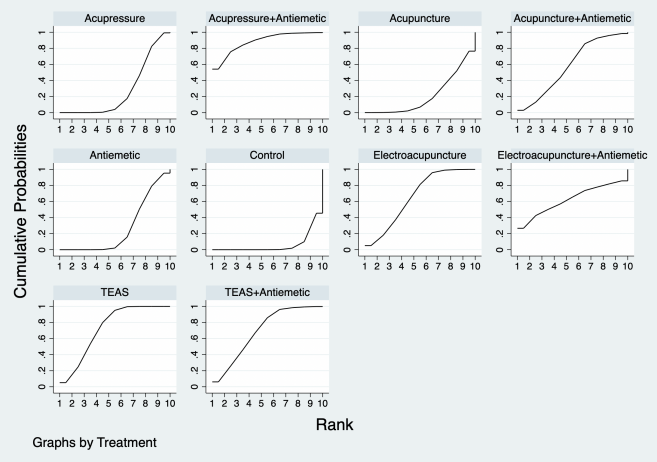


**Figure S7: Funnel diagram(Incidence of PON)**


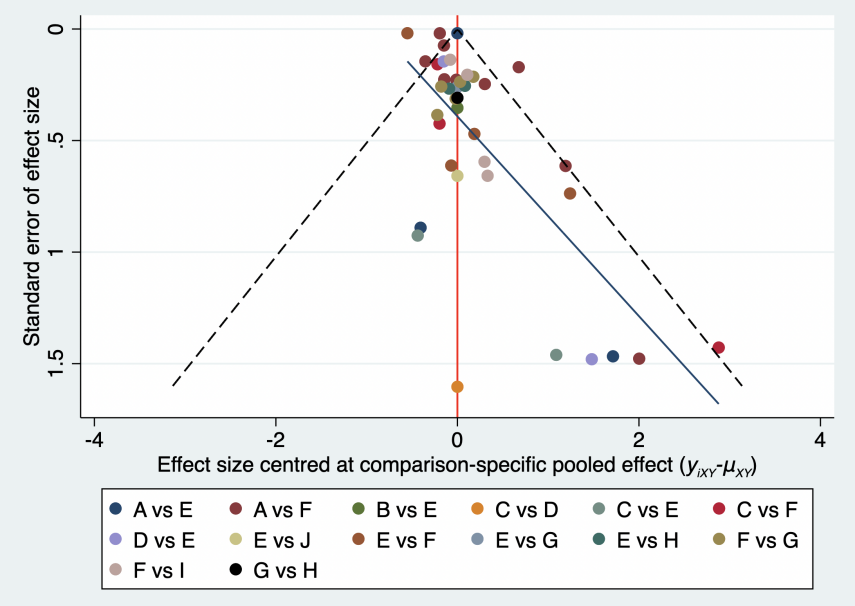


**Figure S8: Forest plot and the global inconsistency test result (Incidence of POV)**

(chi2=10.96, p=0.204)


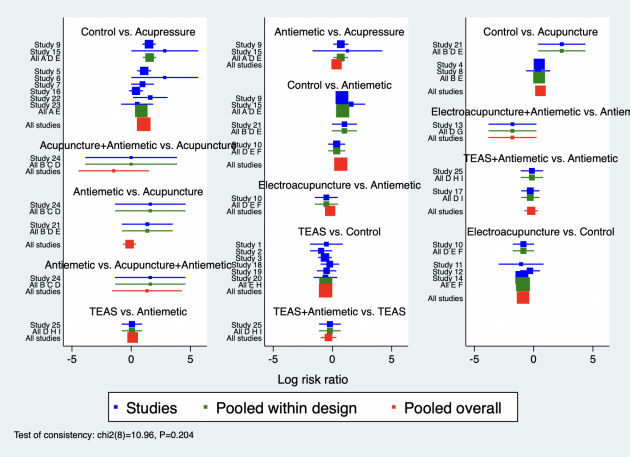


A (reference): Acupressure B: Acupuncture C: Acupuncture+Antiemetic D: Antiemetic E:Control F: Electroacupuncture G: Electroacupuncture+Antiemetic H: TEAS I: TEAS+Antiemetic

**Table S5: Local inconsistency test (Incidence of POV)**


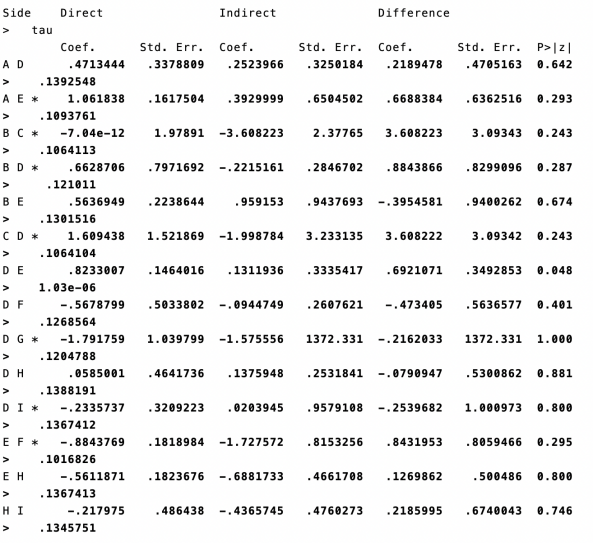


A (reference): Acupressure B: Acupuncture C: Acupuncture+Antiemetic D: Antiemetic E:Control F: Electroacupuncture G: Electroacupuncture+Antiemetic H: TEAS I: TEAS+Antiemetic

**Table S6: SUCRA analysis: MeanRank table (Incidence of POV)**


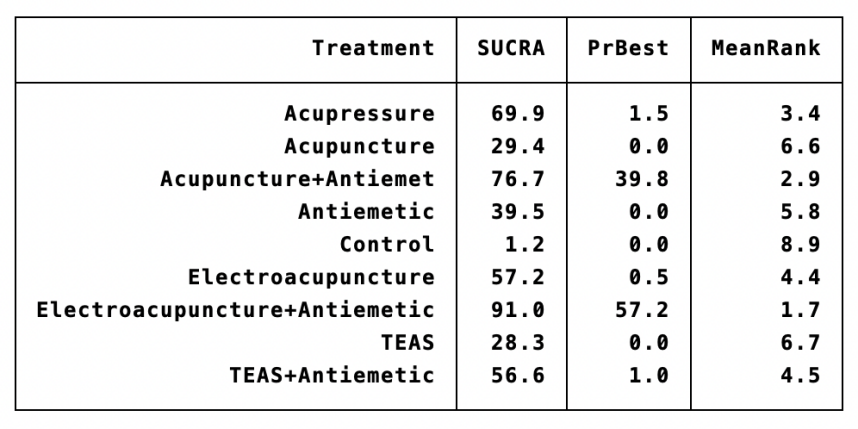


**Figure S9: SUCRA analysis: MeanRank figure (Incidence of POV)**


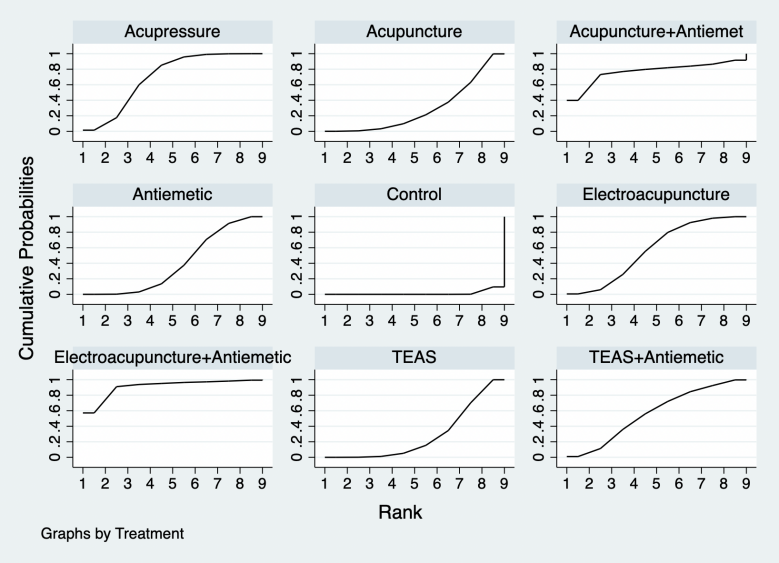


**Figure S10: Funnel diagram(Incidence of POV)**


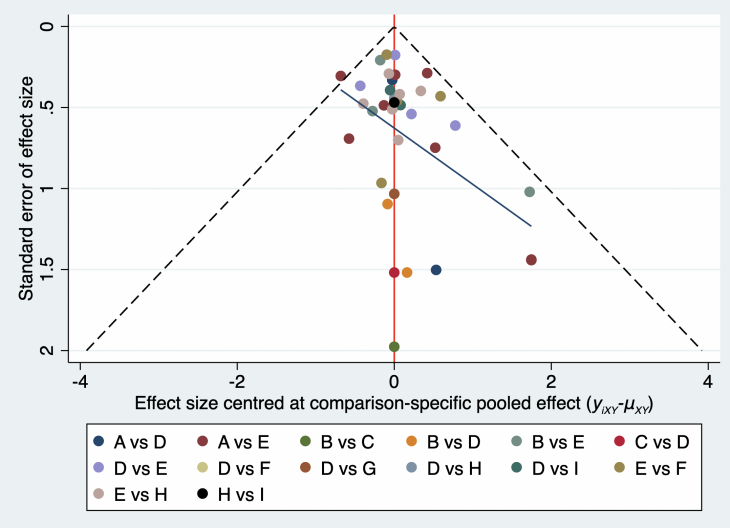


A (reference): Acupressure B: Acupuncture C: Acupuncture+Antiemetic D: Antiemetic E:Control F: Electroacupuncture G: Electroacupuncture+Antiemetic H: TEAS I: TEAS+Antiemetic

**Figure S11: Forest plot and the global inconsistency test result (Number of patients needing antiemetic rescue)**

(chi2=8.11, p=0.088)


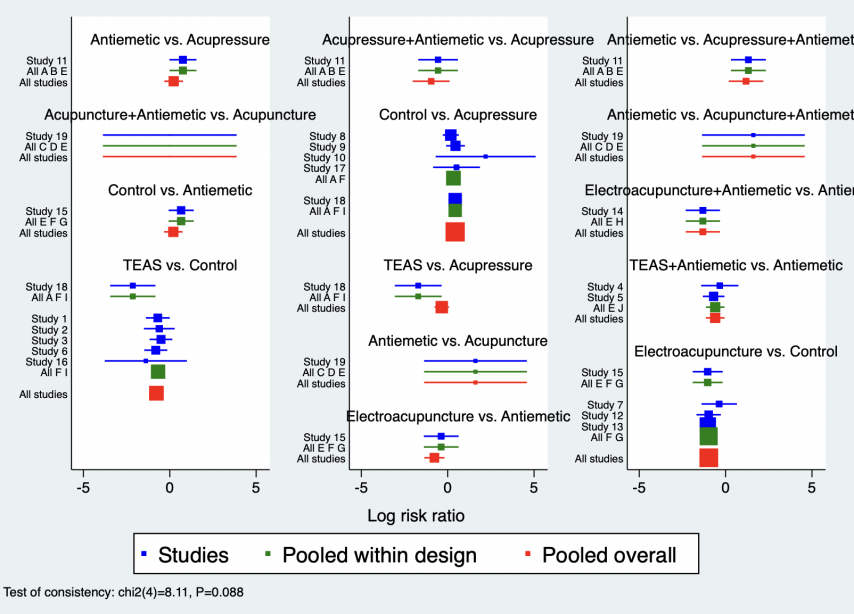


A: Acupressure B: Acupressure+Antiemetic C: Acupuncture D: Acupuncture+Antiemetic E: Antiemetic F: Control G: Electroacupuncture H: Electroacupuncture+Antiemetic I: TEAS J: TEAS+Antiemetic

**Table S7: Local inconsistency test (Number of patients needing antiemetic rescue)**


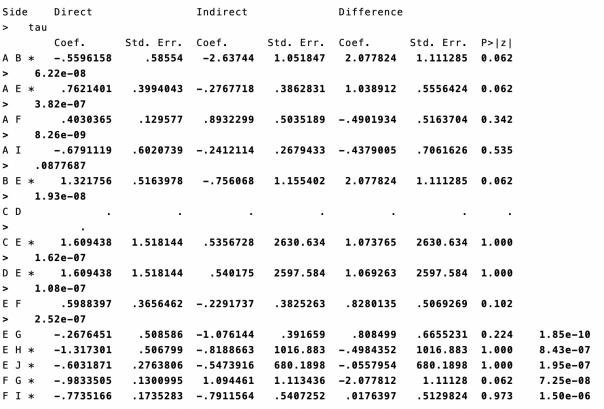


A: Acupressure B: Acupressure+Antiemetic C: Acupuncture D: Acupuncture+Antiemetic E: Antiemetic F: Control G: Electroacupuncture H: Electroacupuncture+Antiemetic I: TEAS J: TEAS+Antiemetic

**Table S8: SUCRA analysis: MeanRank table (Number of patients needing antiemetic rescue)**


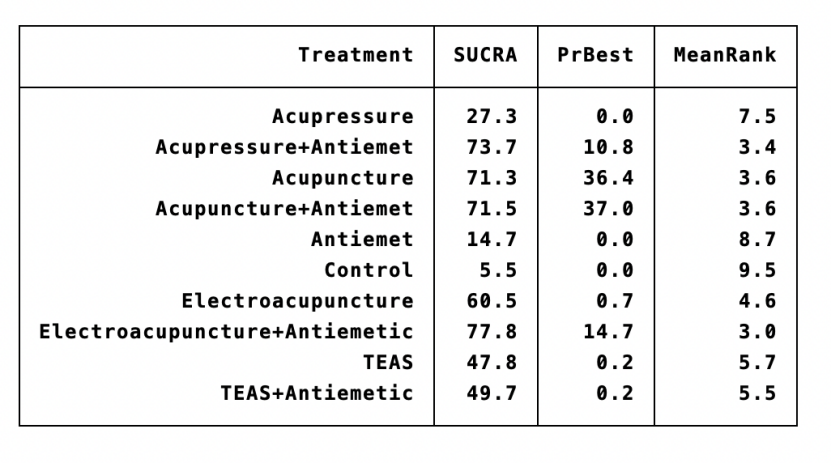


**Figure S12: SUCRA analysis: MeanRank figure (Number of patients needing antiemetic rescue)**


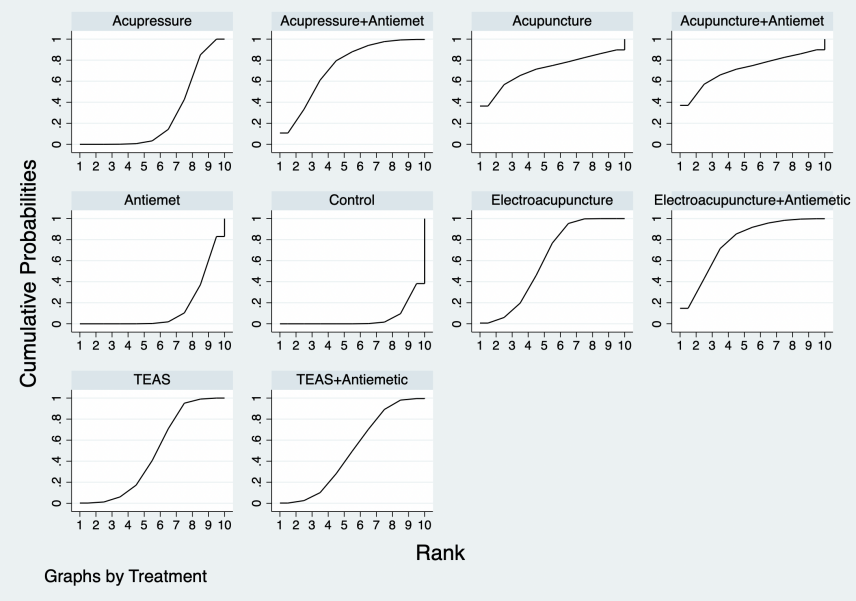


**Figure S13: Funnel diagram(Number of patients needing antiemetic rescue)**


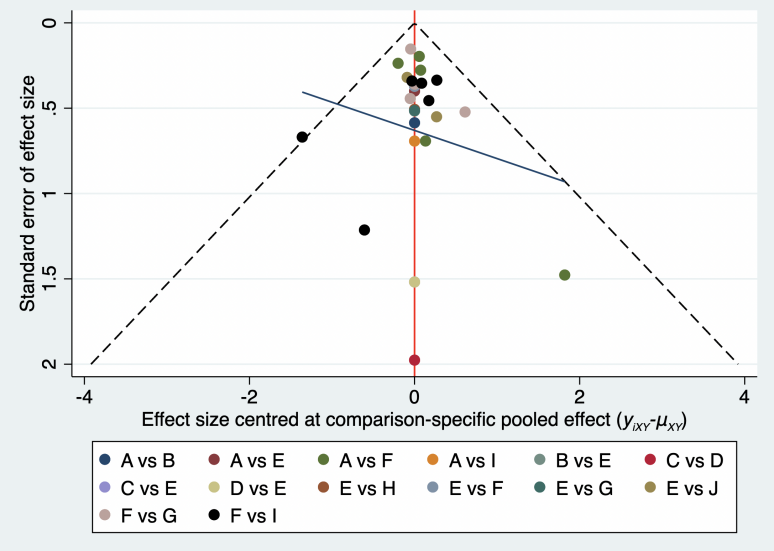


A: Acupressure B: Acupressure+Antiemetic C: Acupuncture D: Acupuncture+Antiemetic E: Antiemetic F: Control G: Electroacupuncture H: Electroacupuncture+Antiemetic I: TEAS J: TEAS+Antiemetic
